# Primary care clinical provider knowledge and experiences in the diagnosis and treatment of tick-borne illness: a qualitative assessment from a Lyme disease endemic community

**DOI:** 10.1186/s12879-021-06622-6

**Published:** 2021-08-31

**Authors:** Stephanie Mattoon, Caitlin Baumhart, Ana C. Barsallo Cochez, Douglas MacQueen, Jeffrey Snedeker, Caroline B. Yancey, Melissa Gatch, Emily M. Mader

**Affiliations:** 1 Department of Population Medicine and Diagnostic Sciences, Cornell University, 618 Tower Road, Ithaca, NY 14853 USA; 2 Environment, Health and Safety, Cornell University, 395 Pine Tree Road, Suite 210, Ithaca, NY 14850 USA; 3 Cayuga Center for Infectious Diseases, 1301 Trumansburg Road, Suite 6, Ithaca, NY 14850 USA; 4 Northeast Pediatrics and Adolescent Medicine, 10 Graham Road West, Ithaca, NY 14850 USA; 5 Tompkins County Health Department, 55 Brown Road, Ithaca, NY 14850 USA; 6 Department of Entomology, Cornell University, 2126 Comstock Hall, Ithaca, NY 14853 USA; 7 Present Address: Center for International Health, Education, and Biosecurity, Institute of Human Virology, University of Maryland Baltimore School of Medicine, Baltimore, USA; 8 Present Address: Pan American Health Organization, Ancón, Avenida Gorgas, Building 261, Panama, Panamá

**Keywords:** Tick-borne disease, Lyme disease, Clinician, Clinician–patient communication, Focus group, Qualitative

## Abstract

**Background:**

Primary care and frontline healthcare providers are often the first point of contact for patients experiencing tick-borne disease (TBD) but face challenges when recognizing and diagnosing these diseases. The specific aim of this study was to gain a qualitative understanding of frontline and primary care providers’ knowledge and practices for identifying TBDs in patients.

**Methods:**

From fall 2018 to spring 2019, three focus groups were conducted with primary care providers practicing in a small-town community endemic to Lyme disease (LD) and with emerging incidence of additional TBDs. A follow up online survey was distributed to urgent and emergency care providers in the small-town community and an academic medical center within the referral network of the local clinical community in spring and summer 2019. Qualitative analysis of focus group data was performed following a grounded theory approach and survey responses were analyzed through the calculation of descriptive statistics.

**Results:**

Fourteen clinicians from three primary care practices participated in focus groups, and 24 urgent and emergency care clinicians completed the survey questionnaire. Four overarching themes emerged from focus group data which were corroborated by survey data. Themes highlighted a moderate level of awareness on diagnosis and treatment of LD among participants and limited knowledge of diagnosis and treatment for two other regionally relevant TBDs, anaplasmosis and babesiosis. Providers described challenges and frustrations in counseling patients with strong preconceptions of LD diagnosis and treatment in the context of chronic infection. Providers desired additional point-of-care resources to facilitate patient education and correct misinformation on the diagnosis and treatment of TBDs.

**Conclusions:**

Through this small study, it appears that clinicians in the small-town and academic medical center settings are experiencing uncertainties related to TBD recognition, diagnosis, and patient communication. These findings can inform the development of point-of-care resources to aid in patient-provider communication regarding TBDs and inform the development of continuing medical education programs for frontline and primary care providers.

**Supplementary Information:**

The online version contains supplementary material available at 10.1186/s12879-021-06622-6.

## Background

Tick-borne diseases (TBDs) are a group of infectious diseases caused by a variety of pathogens transmitted through the bite of an infected tick. The Centers for Disease Control and Prevention (CDC) have identified eight species of hard tick responsible for transmitting multiple bacterial, viral, and protozoan pathogens detrimental to human health in the United States [1, 2]. In the period of 2004–2016, TBDs accounted for over 75% of vector-borne disease reports in the United States, the majority of which being Lyme disease. During this same period, additional TBDs, including spotted fevers, babesiosis, and anaplasmosis, have become increasingly prevalent [1].

Primary care and urgent care clinicians are often the first point of contact for patients suffering from TBDs [3, 4]. However, lack of dedicated clinical training on TBDs can result in uncertainties and challenges when treating patients, as the symptoms of various TBDs can be overlapping, nonspecific, and are often unrecognized [5, 6]. Clinicians are responsible for recognizing, diagnosing, and educating patients on infectious diseases, including tick-borne disease. Incorrect diagnosis or delays in treatments may lead to disease complications and increased healthcare costs [3, 5, 6]. Medical education and residency training programs often provide little instruction regarding TBDs in most specialties and disciplines. The 2020 Health and Human Services Tickborne Disease Working Group Report called for an increase in education on TBDs for the clinical audience [6]. However, limited research has been conducted on optimal continuing medical education (CME) content, methods, and resources for practicing clinicians regarding TBD diagnosis, treatment, and patient communication [7].

The Northeast Regional Center for Excellence in Vector-Borne Diseases (NEVBD) fosters research and education on diseases spread by mosquitoes and ticks in the Northeastern United States. NEVBD collaborated with a local health department, community hospital system, and the Cornell Master of Public Health program, to directly engage with primary care and urgent care clinicians on TBD education in an area endemic to Lyme and other TBDs. The purpose of this effort was to gain a qualitative understanding of the knowledge and experiences of frontline providers in diagnosing and treating TBDs to inform the development of training and resources for this community. Current evidence on provider knowledge and practice regarding the diagnosis and treatment of TBDs has been gained through survey research, and medical chart and billing reviews [7–10]. While these efforts have provided valuable insight into gaps in physician training and practice, they do not provide nuanced details of the thoughts, challenges, and needs of providers as they recognize and treat these diseases during clinical patient encounters. Our work addresses this gap in understanding through a small-scale qualitative-quantitative mixed methods study using focus groups and online surveys.

## Methods

### Focus group data collection and analysis

The initial target study population included primary care and emergency and urgent (frontline) healthcare providers practicing in a small-town community endemic to Lyme disease with emerging incidence of additional TBDs. Invitation emails for focus group participation were sent to practice managers and medical directors for medical practices employing members of the study target population. Researchers coordinated with these key contacts within the practices to schedule in-person focus groups at a date and time convenient to the participating providers. Focus groups were scheduled in late fall 2018 and early spring 2019. Participants included both prescribing (physicians, nurse practitioners, physician assistants) and non-prescribing (licensed practical nurse, registered nurse) providers.

Academic project team members developed guided focus group questions covering current knowledge, perceived challenges, and needed resources on tick-borne illness, which were reviewed by physician and health department team members prior to finalization (Additional file 1). Focus groups were hosted at each participating practice’s office location and time-appropriate meals were offered to participants. After receiving verbal informed consent from participants, a project team member trained in qualitative interviewing techniques (EM or SM) led a group discussion based on the guided questions. All focus groups were audio-recorded and transcribed verbatim for analysis. No names or other personally identifiable information were recorded in the transcripts, including the business names of the participating clinics.

Analysis of the focus group transcripts included open coding of the data with identification of emergent themes, following a predominantly grounded theory approach [11]. Focus group analyses were conducted using Atlas.ti (Scientific Software Development GmbH, Version 8). Four members of the project team (EM, SM, CB, ABC) independently coded one transcript, followed by a joint review and consolidation of the code list. Authors EM and SM then coded all transcripts. Discrepancies between the coding schemes were resolved, and the finalized themes and concepts were reviewed by the larger team.

### Survey data collection and analysis

The project team was unsuccessful in efforts to engage frontline clinicians via focus groups during the project period. After discussing barriers with clinic practice managers, our team determined that we would be unable to engage frontline clinicians in focus groups due to variable schedules and constraints among that population. The project team pursued an alternate approach to engage this group through an online survey questionnaire. Two project team members (CB and ABC) developed a survey questionnaire using validated questions from the published literature [3, 12, 13] and focus group data to measure both baseline provider knowledge and the perceptions and experiences of frontline clinicians practicing in the study’s geographic area. Project team physicians reviewed and beta tested the questionnaire prior to finalization. The questionnaire was open for responses from March to May 2019. Participants were invited via email invitation and informational flyers delivered to hospital and urgent care clinics in the small-town community. Despite several recruitment efforts, and a participation incentive in the form of gift cards to local restaurants, the number of responses to the survey in the targeted small-town community fell well short of the project’s goal.

The project team contacted colleagues at an academic medical center within the referral network of the local clinical community for expanded distribution of the questionnaire to this hard-to-reach population. The survey questionnaire was moderately modified to adapt to the academic medical center clinical community by reducing rating scale complexity, informed by additional beta testing by an infectious disease physician at the academic medical center. This version of the questionnaire was distributed through the clinical network of the academic medical center via email and was open for responses from July 1 to August 31, 2019. Due to the limited sample size generated, analysis of survey responses consisted of calculation of descriptive statistics using Microsoft Excel (Microsoft Corporation, Version 2103).

Protocols and procedures for this study involving human subjects were deemed exempt for review under criterion three by the Institutional Review Board of Cornell University, Protocol Numbers: 1806008097 and 1903008648.

## Results

### Respondent demographics

Three primary care practices scheduled focus groups with the study team. These practices represented internal medicine, family medicine, and pediatric medicine. Participants across the three focus groups included eight physicians (MD/DO), five nurse practitioners, and one licensed practical nurse (total of 14 clinicians). Six clinicians completed the survey questionnaire distributed within the small-town community, including three physicians, two physician assistants, and one nurse practitioner. Eighteen clinicians completed the survey questionnaire distributed to the academic medical center, including 14 physicians, two physician assistants, and two nurse practitioners. Table 1 provides a summary of additional demographic characteristics for survey respondents.

**Table 1:** Demographic characteristics of both small-town and academic medical center online survey questionnaire respondents

	Small-town			Academic Medical Center		
	Physician (n = 3)	Physician assistant (n = 2)	Nurse practitioner (n = 1)	Physician (n = 14)	Physician assistant (n = 2)	Nurse practitioner (n = 2)
Specialty						
Family medicine	2	1	1	–	–	–
Emergency medicine	1	1	–	10	1	1
Internal medicine	–	–	–	2	1	1
Pediatric medicine	–	–	–	2	–	–
Practice location						
Hospital	1	–	–	14	2	2
Urgent care	2	1	1	–	–	–
Private practice	–	1	–	–	–	–
Years in practice						
Less than 1	–	–	1	3	–	–
1–5	–	2	–	4	1	1
5–10	2	–	–	2	1	1
10 or more	1	–	–	5	–	–

### Focus group findings

Four overarching themes emerged from analysis of the focus group transcripts: difficulty in diagnosis; challenges presented by patients and combating misinformation; resources and support to improve diagnosis and treatment; and continuing education and clinical proficiency. Table 2 provides an overview of these themes, explanatory subthemes, and exemplary quotations from the focus group data.

**Table 2:** Themes and subthemes identified from focus group data

Theme	Explanatory subtheme	Exemplary quote
Difficulty in diagnosis	Differential diagnoses are complicated by nonspecific symptoms and interpretation of laboratory results	"…the Lyme test maybe came up with two bands or something like that, three bands, but not enough to confirm that this is Lyme. Like, what do you do? Do you treat the fatigue? Do not treat the fatigue? Everything else is normal." [FG3]
	Clinicians have a self-professed lack of awareness of TBDs outside of Lyme disease	"…I think probably the non-Lyme tick-borne illnesses, which aren’t as readily prevalent, probably aren’t thought of as much as they probably should be." [FG2]
	Lack of standardized diagnosis and treatment practice patterns across specialties	"….we’ve had a number of patients present with effusions to the emergency room and before anybody thinks of Lyme disease, they get their joints tapped." [FG3] “Everybody seems to do it a little bit differently. You get, ‘Well, my other son had it four months ago and that doctor did it this way, and now you’re telling me that way.’”[FG3]
	Presence of EM rash is a key diagnostic feature for frontline providers	"If there’s a bull’s eye rash…we are very clear [in] what we need to do.” [FG3] “If I have somebody come with a bull’s eye, I don’t order a Lyme titer. I just treat them. I think most people would do that anyway.”[FG3]
Challenges presented by patients and misinformation	Educating patients can be more difficult when patients have existing relationships with outside providers	"They have something like chronic fatigue and…they will end up going to one of these…Lyme experts that all they do all day long is put people on something unusual, very strange therapy….And I never know what to do." [FG3]
	Patients often challenge the diagnostic process and recommended treatment plan presented by their provider	"I think patients don’t understand, in a basic way, the uncertainty… there’s uncertainty at every step. They expect black and white answers, and it just isn’t." [FG2]
	Some patients self-diagnose and identify with chronic Lyme disease when experiencing non-specific symptoms	"…some people that come identify with the diagnosis, and you can’t pry them loose." [FG2]
Resources and support to improve diagnosis and treatment of patients	Existing resources do not meet patient accessibility needs	"…the patient population I was working with had a very low literacy level. So, we were looking at a reading level of grade school. It was hard to find resources that were anywhere near approachable for them." [FG1]
	Resources are needed for educating skeptical patients	"A lot of patients have fear of missing it and it becoming debilitating. A lot of that is based on they know someone who has chronic Lyme. So just some factual information about, does chronic Lyme exist? You know, something to provide to the patient." [FG1]
	Access to infectious disease specialists for patient consults and recommendations is helpful	"…If I'm getting a lot of pushback or I'm just not sure, you know, it's just confusing for me, I may have sent a couple of people to [ID doctor] just because he is the ID expert." [FG3]
	Point-of-care, updated reference material is essential	"One of the problems with UpToDate is if you want to click on a photo, all you get is one photo…I mean, you can go on Google and check images and pick up all kinds of stuff, but it will include people with, like stuff that’s not particularly helpful." [FG2] "I mean, again, when you pull them up, you often see 2014, 2015. You know, I like to look and see how recent it is."[FG3]
Continuing education and clinician proficiency	Clinicians need more resources on treatment modalities for different populations	"One dose of doxy[cycline] might be a good prophylaxis, but one dose of amoxicillin isn’t. But people that don’t know ped[iatric]s very well assume, well, if we have to use amoxicillin then we will just do that one dose of amoxicillin. But you can’t do that." [FG3]
	Current training program formats are not conducive to healthcare provider education	"I don’t think just inviting us to a random webinar –we get invited every two days to some drug or some whatever in emails, and I just keep deleting them." [FG3]
	Local epidemiological data and information about tick vectors can directly inform diagnosis and treatment	"I did this tick panel and she came in two weeks later, she had anaplasmosis. But, that was like, whoa. Where did that come from? But, knowing what’s in the community is very useful." [FG2]
	There is a consistent need for education on TBD basics, covering presentation, diagnosis, and treatment	"I mean, most providers have some basic knowledge and don’t have to go back to square one, but there's new information all the time." [FG3]

### Theme 1: Difficulty in diagnosis

Clinicians described multiple challenges in the diagnostic process for TBDs. Clinicians from each of the three participating practices felt that differential diagnoses for TBDs were complicated by the nonspecific symptoms associated with these diseases. Clinicians had a self-professed lack of awareness of TBDs outside of Lyme disease, noting that they were unfamiliar with the signs, symptoms, and appropriate serologic testing needed to diagnose non-Lyme TBDs, as well as the prevalence of these TBDs in their local communities.

Focus group participants reported confidence in diagnosing Lyme disease and developing treatment plans when serologic test results and/or empiric assessment of the patient was conclusive, particularly when the erythema migrans (EM) lesion, or rash, was present. However, interpreting test results for Lyme disease was more difficult in scenarios where patients had nonspecific symptoms in the absence of the characteristic EM rash. At least one participant from each focus group described patients associating nonspecific symptoms, such as fatigue, with Lyme disease specifically and expressed uncertainty around interpreting serology results for Lyme disease. This juxtaposition of nonspecific symptoms and unclear diagnostic testing results proved challenging for clinicians making decisions on how to treat and diagnose their patients for Lyme disease.

“The most annoying one, for me, is the Lyme…part of the differential, but not high on the list, and what do I do about it? So, I’ve got a fever and a little bit of a cough, and my aches, my joints hurt, um, and I live in [endemic area]...So that’s, that actually, it’s frustrating.” [FG1, Family Medicine]

“That’s where I have the hardest time deciphering...when we know in the literature they keep telling us this is a clinical disease, well, here’s a clinical symptom. But, is this Lyme or is it not?” [FG3, Pediatrics]

Another common challenge cited by focus group participants centered on interactions with clinicians from different specialties who had alternative and, at times, conflicting practice patterns for diagnosing and treating TBDs. Focus group participants from the pediatric practice consistently described challenges when patients visited their office following an emergency or urgent care visit related to their TBD due to differences in the diagnostic approaches taken between providers in these two medical specialties. For example, the pediatric providers stated that their first approach to the assessment of swollen joints with no history of trauma is to conduct non-invasive diagnostic testing for Lyme disease. Conversely, these providers felt that patients presenting to the urgent care setting with the same symptoms often received more invasive joint aspiration. Additionally, providers from this practice felt that urgent care and emergency medicine clinicians often prescribed treatments that did not follow established guidelines for tick bite prophylaxis.

"I'll have people come in for a follow up or from seeing like urgent care... and they've put them on one dose of amoxicillin. And I'm like... the literature doesn't show that amoxicillin can be used preventively, you know...Especially I think it's urgent care."[FG3, Pediatrics]

### Theme 2: Challenges presented by patients and misinformation

Focus group participants highlighted challenges in the patient-provider communication dynamic that stemmed from patient orientations toward Lyme disease diagnosis and treatment, competing care plans from outside providers, and the need to address misinformation on TBDs during clinical encounters. Clinicians described the process of redressing misinformation as difficult when patients were attached to specific beliefs and did not accept ‘new’ information discussed during the clinical encounter. In several instances, participants described patients referencing materials on Lyme disease that were counter to the guideline-based resources and evidence they personally rely upon in their medical practice. The patient-provider relationship was at times negatively affected during these interactions.

“I think when people have bad data, that’s particularly hard because no matter what I say, they’re going to be like ‘You’re not Lyme literate. I have other sources.’” [FG1, Family Medicine]

Patient education was more difficult when patients had existing relationships with outside healthcare providers delivering contradictory information. Two focus group discussions centered around differences in care plans between participants’ primary care offices and what they termed the ‘Lyme literate’ community. These participants noted a strong disagreement with the care delivered by these outside providers. In these scenarios of conflict, participants in the family medicine focus group identified infectious disease specialists as a resource of authority to whom they could refer patients. However, providers in the other two focus groups expressed a resigned view of the issue as something they could no longer effectively address within the clinical encounter without damaging the patient-provider dynamic.

“The toughest one I ever had was a young kid who came in with his parents, just for a general checkup, but they mentioned his past history of Lyme disease and how he was being treated at a clinic...for chronic Lyme disease with sequential courses of multiple intravenous antibiotics over and over and over again. And I was just sitting there biting my tongue the whole time. And his complaints were fatigue.[chuckle/sigh]” [FG2, Internal Medicine]

Discussion around the subject of chronic Lyme disease occurred within all three focus groups and coincided with sentiments of frustration. Clinicians described patients who self-diagnose or otherwise identify with the diagnosis of chronic Lyme disease as particularly challenging in both the disease diagnostic process as well as treatment. Clinical encounters with these patients were described as time consuming and difficult. Participants in all three focus groups described experiences where they provided clinical information contradictory to their patients’ pre-existing ideas regarding the biology of Lyme disease infection, resulting in those patients challenging the proposed treatment plans. This commonly came in the form of patients rejecting results of serologic testing, wanting a different length of antibiotic course than that prescribed, or seeking an alternative treatment modality.

### Theme 3: Resources and support to improve diagnosis and treatment of patients

Clinicians participating in the focus groups largely felt that existing resources to aid with patient education did not meet their needs. Clinicians felt their ability to disseminate information to patients was influenced by patient education level, and there was sentiment among participants that resources for the lay audience on Lyme and other TBDs are limited. Clinicians also described wanting point-of-care resources for patients, either through flyers, pamphlets, or a reference list of online resources. Ideally, this educational material would support clinicians' recommendations, help to minimize patient doubt, and support trust in the patient-provider relationship. Participants in two focus groups specifically described wanting resources that directly address misinformation and myths around chronic Lyme disease using evidence-based information.

When describing reference materials to inform their clinical practice, focus group participants emphasized that the resources need to be both available at the point-of-care and up to date. Participants in two focus groups described using UpToDate.com as their primary source for both obtaining information for their clinical practice and to give to patients. Participants in each focus group felt that access to infectious disease specialists for patient consults and recommendations was helpful for their practice and often referred patients to infectious disease specialists when diagnoses were unclear.

### Theme 4: Continuing education and clinical proficiency

Participants in all three focus groups felt that the predominant web-based format for CME is not accessible or conducive to their education. Specific suggestions on effective training included interactive instruction and review of case studies.

“To have someone be able to answer questions online while you’re watching the presentation because often there are a lot of questions, which don’t have the time to get answered…A little bit more case studies...because that’s what a lot of the primary care doctors are facing.” [FG1, Family Medicine]

Specific content areas to include in CME programs included resources and information on treatment modalities for unique populations, including pediatrics and individuals with antibiotic allergies; local epidemiological data and information on tick vectors; and updates on TBD basics including presentation, diagnostics, and treatment. Clinicians felt resources that included case studies and images of EM rashes would improve their ability to diagnose TBDs. Data from the focus group transcripts also identified an issue regarding diagnostic testing for TBDs related specifically to appropriate test selection in the electronic medical record (EMR). Clinicians expressed a lack of knowledge on differentiating test options in the EMR and their appropriateness for Lyme disease diagnosis. Clinicians may not be familiar with the differences between available tests and, when given multiple options, make inappropriate selections. Clinicians felt additional training and guidance in this area would be beneficial.

"I ordered what I thought was sort of the standard Lyme titer. It came back negative and this kid continued to have a swollen knee...He went to the orthopedist. They couldn't figure it out. He's going up to the rheumatologist. And it's just because I ordered the wrong test...I think [I] ordered the PCR, which was like, you know, the quick and easy one. But actually that wasn't probably a good one to do." [FG3, Pediatrics]

### Survey findings

Approximately half of survey respondents self-rated as moderately knowledgeable on the treatment of anaplasmosis, babesiosis, and Lyme disease (Table 3). While a sizeable proportion reported feeling not at all knowledgeable on anaplasmosis (45.8%), roughly the same proportion reported feeling extremely knowledgeable on the treatment of Lyme disease. When presented with a clinical scenario and list of treatment options, the majority of respondents selected the CDC-recommended treatment approach for each of the indicated TBDs. However, respondents appeared to have difficulty in identifying the appropriate treatment approach for patients with nonspecific symptoms and negative Lyme disease serology, with only 66.7% providing the correct answer (See Additional file 2 for full question item text).

**Table 3:** Combined self-reported and measured knowledge on the clinical management of anaplasmosis, babesiosis, and Lyme disease for small-town and academic medical center clinicians

	Self-reported knowledge on clinical management of TBD			Correct TBD treatment selected^a^
	Not at all knowledgeable	Moderately knowledgeable	Extremely knowledgeable	
Anaplasmosis	11 (45.8%)	12 (50.0%)	1 (4.2%)	20 (83.3%)
Babesiosis	9 (37.5%)	15 (62.5%)	0 (0.0%)	12 (50.0%)
Lyme Disease	0 (0.0%)	13 (54.2%)	11 (45.8%)	24 (100%) EM rash
				16 (66.7%) Negative serology^b^
				18 (75.0%) Positive serology^b^

^a^Correct response corresponds to selection of CDC recommended treatment for indicated TBD from multiple choice question item

^b^Patient with a 3-month history of recurrent, asymmetric arthritis involving large, weight-bearing joints; no history of erythema migrans; unknown tick bite history; outdoor enthusiast

Half of the respondents reported feeling neutral on their ability to address misinformation on TBDs and misinformation on Lyme disease specifically, while 37.5% and 41.7% felt confident in their ability to address misinformation on TBDs and Lyme disease, respectively. Most respondents (91.7%) reported that patients rarely or never refused to take the antibiotic treatment they prescribe to treat TBDs. Moreover, 37.5% of respondents reported that, about half of the time, patients request a longer course of antibiotic treatment, and 45.8% reported patients try to negotiate on the length of antibiotic treatment to treat TBDs.

Half the respondents reported using educational tools to help their patients better understand TBDs, but only 41.7% reported that current resources for patient education are sufficient and 83.3% reported wanting additional educational resources for their patients on TBDs. Respondents indicated they often use information on UpToDate.com, the CDC website, and the CDC tick-borne disease handbook [2] to educate their patients on TBDs. In written responses on why these materials were their preferred resources, respondents indicated they were readily accessible, easy to understand, and accurate.

The majority of respondents (79.2%) reported they have access to the resources they need to update their personal knowledge on TBDs, and close to half of respondents (45.8%) reported regularly looking up peer-reviewed literature on the treatment and diagnosis of TBDs. All respondents reported sometimes or often using UpToDate.com to access information on TBDs. Other common resources respondents used (sometimes or often) included the CDC website (79.2%), medical journals (66.7%), and Infectious Disease Society of American (IDSA) guidelines (54.2%). Just over half of respondents (55.6%) reported never attending CME accredited seminars, and 88.9% reported never watching CME accredited webinars to learn about TBDs. Additional summaries on survey response distributions are available in Additional file 2.

## Discussion

From our small study in a Lyme disease endemic community, it appears that primary care and frontline healthcare providers experience uncertainty in the diagnosis and clinical management of TBDs. The first focus group theme we identified indicates that primary care clinicians lack confidence in their ability to diagnose and treat non-Lyme TBDs and to identify Lyme disease infection in ambiguous clinical scenarios. Participant statements reflected several points of frustration related to this uncertainty. Frustration in the decision-making process in the context of inconclusive Lyme disease serology centered not only on the clinicians’ internal misgivings about their own knowledge, but also on how this uncertainty resulted in lengthy and difficult conversations with patients. The study data also indicate that the approach to Lyme disease diagnosis and treatment is variable across medical specialties. Inconsistency in practice patterns and variable adherence to guideline-based care across healthcare providers negatively affected patient trust, further complicating patient education, counseling, and care plan development.

Another prevalent focus group theme focused on the contentious issue of chronic Lyme disease and conflicting guidance from alternative care providers. Participants frequently mentioned conversations with patients on this topic, consistently reporting the need to address misinformation on Lyme disease during the patient encounter. Participants often felt frustrated about this misinformation, knowing it came from sources they did not regard as authoritative or guideline-based, including word of mouth, social media, and alternative “Lyme literate” care providers. As stated by the National Institutes of Health, “chronic Lyme disease” is not a universally recognized disease due to confusion on diagnosis and the lack of a clear clinical definition [14]. Several clinicians described a shared experience of feeling helpless to correct misinformation when confronted with strongly held patient beliefs and anxieties regarding persistent Lyme disease infection, at the risk of alienating their patients. This introduced a reliance on consultation and referral to infectious disease physicians to assist in managing care for these patients.

While only 24 clinicians participated in our survey questionnaire, their responses reflect certain findings from the focus group data. Roughly one-third of respondents failed to identify the CDC-recommended treatment approach for patients with nonspecific symptoms and negative Lyme disease serology, reflecting a similar challenge identified through the focus group data. While survey respondents reported feeling confident in their ability to address misinformation on TBDs with their patients, close to half reported receiving push back from patients on the recommended treatment regimen. While our survey sample size is too small to draw broad conclusions or generalize to larger populations, these novel question items may be useful in future, larger surveys measuring clinician experiences regarding TBD diagnosis and treatment.

We identified opportunities to improve clinician knowledge regarding TBD diagnosis and management, including development of a comprehensive algorithm to standardize care across specialties, providing updated epidemiological data for regional TBDs, and a primer on how to order the appropriate Lyme serology in the EMR. While our study particularly struggled to reach emergency and urgent care specialists, making connections with practicing clinicians overall is challenging [15, 16]. Our data indicate that training formats of CME-based webinars or seminars are not useful for reaching these populations. Unfortunately, many of the existing training formats on Lyme and other TBDs fall within these categories [17–19]. Results from both the focus groups and survey questionnaires indicate that providers gather information for themselves and for their patients through trusted point-of-care resources, specifically UpToDate.com. Future efforts to provide resources on TBDs for healthcare providers should take into consideration where and how this information will be accessed. In addition, resources developed with shared decision-making and clinical decision support incorporated have demonstrated positive effects on patient-provider communication in other areas of healthcare [6, 20–23]. Similar resource development targeting TBDs may prove beneficial for challenging scenarios described by clinicians in this study.

### Limitations

One issue we encountered in focus group recruitment for this study was a hesitancy of practices to participate due to differing orientations of medical staff on the issue of chronic Lyme disease. In cases where practices included clinical staff with strong and discordant beliefs on diagnosis and treatment of chronic Lyme disease, these focus groups were perceived as harmful to office dynamics. Medical directors at two practices declined to organize focus groups at their practices due to this contentious issue. While we did not specifically ask focus group participants to identify their opinions on this issue, several of our participants openly stated they did not believe in the chronic Lyme disease diagnosis. Thus, our focus group findings do not include the experiences of alternative care providers or providers who follow International Lyme and Associated Diseases Society (ILADS) guidelines.

An additional issue encountered in this project was the low response rate from the emergency and urgent care clinical community, for both the focus group interviews and the online survey questionnaire. Our low response rates limit the generalizability of our findings beyond the small group of study participants. Frontline providers in the emergency and urgent care clinical community play an important role in diagnosing and treating TBDs, and additional efforts are needed to engage with this hard-to-reach community. Additionally, during the survey, clinician’s knowledge was tested on CDC-recommended treatments for specific TBDs. Due to the survey being online, it is possible that the high-performance scores were due to respondents looking up the correct answers.

## Conclusion

The gaps in knowledge identified through the focus group and online survey data, coupled with the consistent necessity to provide point-of-care counseling and education to patients, highlight a pressing need for resources and support for primary care and frontline providers treating patients for TBDs. Participants from both the focus groups and survey questionnaire indicated a desire for more educational resources to share with patients on TBDs. Focus group participants specifically described these resources as tools to support productive conversations with patients on TBDs in a wide range of subject matter, including explaining the diagnosis process, explaining the evidence behind treatment, and dispelling misinformation regarding treatment efficacy and persistent infection. Additional efforts are also needed to provide ongoing TBD educational opportunities that are accessible to these hard-to-reach clinical communities.

## Supplementary Information

**Additional file 1.** Focus group and survey questionnaire data collection tools. Full listing of focus group discussion script (primary questions and prompts) and frontline provider survey questionnaire.

**Additional file 2.** Survey responses. Response distributions for all question items included in the frontline provider survey questionnaire.

## Data Availability

Aggregated survey responses are available in the additional files included with this publication. The datasets used and/or analyzed during the current study are available from the corresponding author on reasonable request.

## Abbreviations

CDC: Centers for Disease Control and Prevention
CME: Continuing medical education
DO: Doctor of osteopathic medicine
EMR: Electronic medical record
FG: Focus group
ID: Infectious disease
IDSA: Infectious Diseases Society of America
ILADS: International Lyme and Associated Diseases Society
LD: Lyme disease
MD: Medical doctor
NEVBD: Northeast Regional Center for Excellence in Vector-Borne Diseases
TBD: Tick-borne disease
