# Supplementary material for: Primary care clinical provider knowledge and experiences in the diagnosis and treatment of tick-borne illness: a qualitative assessment from a Lyme disease endemic community
Source: BMC Infect Dis. 2021 Aug 31;21:894. doi: 10.1186/s12879-021-06622-6 (PMC8408947; doi:10.1186/s12879-021-06622-6)
Supplement: Supplementary file 1 — Additional file 1. Focus group and survey questionnaire data collection tools. Full listing of focus group discussion script (primary questions and prompts) and frontline provider survey questionnaire. [file 12879_2021_6622_MOESM1_ESM.docx]

**Additional File 1: Focus Group and Survey Questionnaire Data Collection Tools**

**Focus Group Discussion Questions**

*Ice breaker questions:*

1. Can one or two of you share a story about a challenging situation with a TBD?

*Management of Tick-borne Disease*

1. How comfortable do you feel diagnosing and treating tick-borne illnesses with your patients?
   1. What are challenges you face around using diagnostic tools for TBDs?
   2. Do you feel confident diagnosing TBD’s other than Lyme (anaplasmosis, babesiosis, ehrlichiosis, RMSF)?
   3. What uncertainties do you have when choosing a treatment plan?

*Client communication*

1. What are the most challenging patient TBD questions for you to answer?
   1. What resources do you think would make these conversations less challenging?
2. What sorts of patient educational materials and resources do you have for tick-borne disease prevention and treatment?
   1. What are the things you like and dislike about these educational materials?
3. Where do you send patients for more information?

*Training format*

1. What are the key things you would like covered or provided in a tick-borne disease training program?
2. Treatment of TBD’s
3. Diagnosis of TBD’s
4. Are there particular diagnostic tests that you would like to better understand?
5. TBD Prevention methods
6. Client communication techniques for TBD’s
7. Tick life cycle and biology, species of ticks
8. Decision making?
9. Provision of decision support tools (An app? Hardcopies? Posters? Image-based?)
10. Specific TBD’s, new and emerging TBD’s
11. Are there certain patient populations you want to know more about (like young children or immunocompromised patients)?
12. What information sources do you use to learn about tick-borne disease?
13. How often do you use these resources?
14. What are things you like and dislike about these resources?
15. Which do you feel are most reliable and current
16. What method of delivery or approach to a TBD training would be most helpful to you?
17. Case studies?
18. PowerPoint/didactic?
19. Discussion?
20. What would be a feasible length of training?
    1. Is there a particular time of day and day of week that would make it easier for you to attend?
    2. Is there a location that would make it easier for you to attend?
    3. In addition to CME credit, what would encourage you to attend?

**Survey Questionnaire**

*Demographics*

1. What is your discipline?
   1. Doctor of Medicine
   2. Doctor of Osteopathic Medicine
   3. Physician Assistant
   4. Nurse Practitioner
   5. Registered Nurse
   6. Bachelor of Science in Nursing
   7. Other (please specify)
2. Where do you practice most?
   1. Hospital
   2. Urgent Care
   3. Convenient Care
   4. Private Practice
   5. Other (please specify)
3. What is your area of practice?
   1. Emergency Medicine
   2. Internal Medicine
   3. Pediatric Medicine
   4. Family Medicine
   5. Infectious Diseases
   6. Pathology
   7. Other (please specify)
4. How many years have you practiced in your current location?
   1. Less than 1 year
   2. 1 - 5 years
   3. 5 - 10 years
   4. 10 or more years

*Clinical Knowledge*

1. How often did you encounter the following tick-borne diseases in the last 12 months?

|  | Never | Sometimes | Often |
| --- | --- | --- | --- |
| Anaplasmosis |  |  |  |
| Babesiosis |  |  |  |
| Lyme Disease |  |  |  |

1. How knowledgeable do you feel about the **diagnosis** of the following tick-borne diseases?

|  | Not at all knowledgeable | Moderately knowledgeable | Extremely knowledgeable |
| --- | --- | --- | --- |
| Anaplasmosis |  |  |  |
| Babesiosis |  |  |  |
| Lyme Disease |  |  |  |

1. How comfortable are you diagnosing Lyme disease when **no erythema migrans rash** is present?
   1. Not at all comfortable
   2. Moderately comfortable
   3. Extremely comfortable
2. Which of the following tests for Lyme disease have you ordered in the past 12 months? (select all that apply)
   1. Serum ELISA
   2. Serum Western blot
   3. C6 Assay
   4. Polymerase chain reaction (PCR)
   5. Urine antigen test
   6. Any test of CSF
   7. Other (please specify)
   8. N/A - I have not ordered any tests for Lyme disease in this period
3. How knowledgeable do you feel about the **clinical management** of the following tick-borne diseases?

|  | Not at all knowledgeable | Moderately knowledgeable | Extremely knowledgeable |
| --- | --- | --- | --- |
| Anaplasmosis |  |  |  |
| Babesiosis |  |  |  |
| Lyme Disease |  |  |  |

1. How would you treat the following case: 
   **A patient with Erythema migrans; no laboratory testing performed to date.**
   1. Treat with an antibiotic at this time
   2. No antibiotic at this time; reassure and educate the patient, with no further follow-up
   3. No antibiotic at this time; no treatment or testing now, but see the patient for follow-up
   4. No antibiotic at this time; test patient for Lyme disease
   5. No antibiotic at this time; refer patient to a specialist
2. How would you treat the following case: 
    
   **A patient with a 3 month history of recurrent, asymmetric arthritis involving large, weight-bearing joints. The patient has no history of erythema migrans and has had multiple negative Lyme screen tests (Lyme EIAs) over the past 3 months. Whether the patient has ever been bitten by a black-legged (deer) tick is unknown, but the patient spends a lot of time outdoors. There was no cause for patient's arthritis found on initial work up.**
   1. Treat with an antibiotic at this time
   2. No antibiotic at this time; continue to investigate other possible causes of the arthritis
   3. No antibiotic at this time; refer patient to a specialist
   4. No antibiotic at this time; further testing for Lyme disease now
3. How would you treat the following case: 
    
   **A patient with a 3 month history of recurrent, asymmetric arthritis involving large, weight-bearing joints. The patient has no history of erythema migrans and has had the following over the past 3 months: A positive Lyme screen test (Lyme EIA), a positive WB IgM, and a negative WB IgG, but with a band at p41. Whether the patient has ever been bitten by a black-legged (deer) tick is unknown, but the patient spends a lot of time outdoors. There was no cause for patient's arthritis found on initial work up.**
   1. Treat with an antibiotic at this time
   2. No antibiotic at this time; continue to investigate other possible causes of the arthritis
   3. No antibiotic at this time; refer patient to a specialist
   4. No antibiotic at this time; further testing for Lyme disease now
4. What is the CDC recommended treatment for adults diagnosed with **anaplasmosis**?  Select all that apply:
   1. Doxycycline for 10 -14 days
   2. Atovaquone + azithromycin for 7 -10 days
   3. Clindamycin + quinine for 7-10 days
   4. Amoxicillin for 10-14 days
5. What is the CDC recommended treatment for adults diagnosed with **babesiosis**? Select all that apply:
   1. Doxycycline for 10-14 days
   2. Atovaquone + azithromycin for 7-10 days
   3. Clindamycin + quinine for 7-10 days
   4. Amoxicillin for 10-14 days

*Perceptions and Experiences*

1. To what extent do you agree with the following statements:

|  | Disagree | Neutral | Agree |
| --- | --- | --- | --- |
| I find laboratory results for tick-borne diseases hard to interpret. |  |  |  |
| I find laboratory results hard to interpret for Lyme disease specifically. |  |  |  |
| Patients question my knowledge regarding tick-borne diseases. |  |  |  |
| Patients do not understand me when I explain tick-borne disease diagnosis. |  |  |  |
| Patients do not trust my guidance regarding the treatment of tick-borne diseases. |  |  |  |

1. To what extent do you agree with the following statements:

|  | Disagree | Neutral | Agree |
| --- | --- | --- | --- |
| I am confident in my ability to discuss information about tick-borne diseases with patients. |  |  |  |
| I am confident in my ability to address misinformation about tick-borne diseases. |  |  |  |
| I am confident in my ability to address misinformation about Lyme disease specifically. |  |  |  |

1. How frequently do you encounter the following situations when discussing the use of antibiotics to treat tick-borne disease?

|  | Rarely | About half the time | Most of the time |
| --- | --- | --- | --- |
| Patients refuse to take the antibiotics I prescribe. |  |  |  |
| Patients request a shorter course of antibiotics than I am comfortable giving them. |  |  |  |
| Patients accept the antibiotic treatment plan I give to them. |  |  |  |
| Patients request a longer course of antibiotics than I am comfortable giving them. |  |  |  |
| Patients try to negotiate with me on the length of their antibiotic course. |  |  |  |
| Patients request an alternative medicine form of treatment. |  |  |  |

*Tools and Resources*

1. To what extent do you agree with the following statements:

|  | Disagree | Neutral | Agree |
| --- | --- | --- | --- |
| I use educational tools with my patients to help them better understand tick-borne diseases |  |  |  |
| Current resources available to me for patient education are sufficient |  |  |  |

1. How often do you use the following resources to **educate patients** about tick-borne diseases?

|  | Never | Sometimes | Often |
| --- | --- | --- | --- |
| Resources from the local health department |  |  |  |
| Resources from the state health department |  |  |  |
| US Centers for Disease Control and Prevention website |  |  |  |
| US Centers for Disease Control and Prevention Tick-Borne Disease Handbook |  |  |  |
| Medscape |  |  |  |
| Up to Date |  |  |  |
| Medical or public health journals |  |  |  |
| Other (specify) |  |  |  |

1. What do you like about the patient education materials you use? Select all that apply:
   1. Easy to access
   2. Easy to understand
   3. Accurate information
   4. Regularly updated
   5. Visually appealing
   6. Easy to share with patients
   7. Easy to print
   8. Other (please specify)
   9. N/A - I do not use tick-borne illness patient educational materials
2. Would you like additional educational resources on tick-borne diseases for your patients?
   1. Yes
   2. No

*Display This Question:*

*If Would you like additional educational resources on tick-borne diseases for your patients? = Yes*

1. What format would you prefer for educational resources for patients? Select all that apply:
   1. Posters
   2. Flyers/pamphlets
   3. Online resources
   4. Other (please specify)

*Display This Question:*

*If Would you like additional educational resources on tick-borne diseases for your patients? = Yes*

1. What topics should educational resources for patients to cover?
   1. Types of ticks in our area
   2. How ticks transmit disease
   3. Symptoms of tick-borne disease
   4. Interpreting tests to diagnose tick-borne disease
   5. Tick-borne disease treatment
   6. Efficacy of tick-borne disease treatment
   7. Other (please specify)
2. What do you think would make educational resources for patients more effective?

*Clinical Education Resources and Training Preferences*

1. To what extent do you agree with the following statements:

|  | Disagree | Neutral | Agree |
| --- | --- | --- | --- |
| I regularly look-up or research literature on tick-borne diseases. |  |  |  |
| I have access to the resources I need to update my personal knowledge about tick-borne diseases. |  |  |  |

1. How often do you use the following resources to access information on the management of tick-borne diseases?

|  | Never | Sometimes | Often |
| --- | --- | --- | --- |
| CME-accredited seminars |  |  |  |
| CME-accredited webinars |  |  |  |
| Guidelines from the Infectious Diseases Society of America |  |  |  |
| Guidelines from the International Lyme and Associated Diseases Society |  |  |  |
| Publications in medical journals |  |  |  |
| Centers for Disease Control and Prevention website |  |  |  |
| Centers for Disease Control and Prevention Tick-Borne Disease Handbook |  |  |  |
| National Institutes of Health website |  |  |  |
| Medscape |  |  |  |
| Up to Date |  |  |  |
| Other (please specify): |  |  |  |

1. What is the number one way in which these resources can be improved?
2. How likely are you to participate in a tick-borne disease training course that does **not** carry continuing education credit?
   1. Not likely
   2. Somewhat likely
   3. Very likely
3. Please select the topics that would be **most useful to you** to include in a tick-borne disease training program: (select all that apply)
   1. Patient communication techniques on tick-borne diseases
   2. Tick life cycle and biology
   3. Species of ticks in your community
   4. Epidemiological data for tick-borne diseases in your community
   5. Decision making around clinical management of tick-borne diseases
   6. Use of decision support tools for tick-borne disease treatment
   7. Treatment for at-risk populations (e.g., outdoor workers)
   8. Treatment for specific patients (pediatrics and contraindications)
   9. Other (please specify)
4. Please rank the following training formats by your ability to access them, with the most accessible format ranked 1 and least accessible format ranked 5.
5. In person seminar
6. Printed materials
7. Webinar
8. Online materials
9. Other
10. Please use the space below to share additional feedback regarding tick-borne disease training, communication, or resource needs that were not covered in this survey.
