# Supplementary material for: Primary care clinical provider knowledge and experiences in the diagnosis and treatment of tick-borne illness: a qualitative assessment from a Lyme disease endemic community
Source: BMC Infect Dis. 2021 Aug 31;21:894. doi: 10.1186/s12879-021-06622-6 (PMC8408947; doi:10.1186/s12879-021-06622-6)
Supplement: Supplementary file 2 — Additional file 2. Survey responses. Response distributions for all question items included in the frontline provider survey questionnaire. [file 12879_2021_6622_MOESM2_ESM.docx]

**Additional File 2: Survey responses**

*Calculations were made based on N=24 respondents, unless otherwise indicated.*

***Clinical Knowledge***

Q5. How often did you encounter the following tick-borne diseases in the last 12 months?

| Tick-Borne Illness | Response Options (N, %) | | |
| --- | --- | --- | --- |
|  | Never | Sometimes | Often |
| Anaplasmosis* | 19 (86.4) | 3 (13.6) | 0 (0.0) |
| Babesiosis** | 20 (87.0) | 3 (13.0) | 0 (0.0) |
| Lyme Disease | 1 (4.2) | 9 (37.5) | 14 (58.3) |
| * 2 non-responses, total responses: N=22  **1 non-response, total responses: N= 23 | | | |

Q6. How knowledgeable do you feel about the **diagnosis** of the following tick-borne diseases?

| Tick-Borne Illness | Response Options (N, %) | | |
| --- | --- | --- | --- |
|  | Not at all Knowledgeable | Moderately Knowledgeable | Extremely Knowledgeable |
| Anaplasmosis | 13 (54.2) | 10 (41.7) | 1 (4.2) |
| Babesiosis | 9 (37.5) | 14 (58.3) | 1 (4.2) |
| Lyme Disease | 1 (4.2) | 13 (54.2) | 10 (41.7) |

Q7. How comfortable are you diagnosing Lyme disease when **no erythema migrans rash** is present?

| **Response Option** | **N** | **%** |
| --- | --- | --- |
| Not at all knowledgeable | 2 | 8.3 |
| Moderately knowledgeable | 18 | 75.0 |
| Extremely knowledgeable | 4 | 16.7 |

Q8. Which of the following tests for Lyme disease have you ordered in the past 12 months? (select all that apply)

| **Test** | **N** | **%** |
| --- | --- | --- |
| Serum ELISA | 18 | 75.0 |
| Serum Western blot | 17 | 70.8 |
| C6 Assay | 0 | 0.0 |
| Polymerase chain reaction (PCR) | 11 | 45.8 |
| Urine antigen test | 0 | 0.0 |
| Any test of CSF | 9 | 37.5 |
| Other | 0 | 0.0 |
| None | 0 | 0.0 |

Q9. How knowledgeable do you feel about the **clinical management** of the following tick-borne diseases?

| Tick-Borne Illness | Response Options (N, %) | | |
| --- | --- | --- | --- |
|  | Not at all Knowledgeable | Moderately Knowledgeable | Extremely Knowledgeable |
| Anaplasmosis | 11 (45.8) | 12 (50.0) | 1 (4.2) |
| Babesiosis | 9 (37.5) | 15 (62.5) | 0 (0.0) |
| Lyme Disease | 0 (0.0) | 13 (54.2) | 11 (45.8) |

Q10. How would you treat the following case: **A patient with Erythema migrans; no laboratory testing performed to date.**

| **Response Option** | **N** | **%** |
| --- | --- | --- |
| Treat with an antibiotic at this time** | 24 | 100.0 |
| No antibiotic at this time; reassure and educate the patient, with no further follow-up | 0 | 0.0 |
| No antibiotic at this time; no treatment or testing now, but see the patient for follow-up | 0 | 0.0 |
| No antibiotic at this time; test patient for Lyme disease | 0 | 0.0 |
| No antibiotic at this time; refer patient to a specialist | 0 | 0.0 |

***Correct response*

Q11. How would you treat the following case: **A patient with a 3-month history of recurrent, asymmetric arthritis involving large, weight-bearing joints. The patient has no history of erythema migrans and has had multiple negative Lyme screen tests (Lyme EIAs) over the past 3 months. Whether the patient has ever been bitten by a black-legged (deer) tick is unknown, but the patient spends a lot of time outdoors. There was no cause for patient's arthritis found on initial work up.**

| **Response Option** | **N** | **%** |
| --- | --- | --- |
| Treat with an antibiotic at this time | 5 | 20.8 |
| No antibiotic at this time; continue to investigate other possible causes of the arthritis** | 8 | 33.3 |
| No antibiotic at this time; refer patient to a specialist** | 8 | 33.3 |
| No antibiotic at this time; further testing for Lyme disease now | 3 | 12.5 |
| ***Correct responses: 16 (66.67%) respondents* | | |

Q12. How would you treat the following case: **A patient with a 3-month history of recurrent, asymmetric arthritis involving large, weight-bearing joints. The patient has no history of erythema migrans and has had the following over the past 3 months: A positive Lyme screen test (Lyme EIA), a positive WB IgM, and a negative WB IgG, but with a band at p41. Whether the patient has ever been bitten by a black-legged (deer) tick is unknown, but the patient spends a lot of time outdoors. There was no cause for patient's arthritis found on initial work up.**

| **Response Option** | **N** | **%** |
| --- | --- | --- |
| Treat with an antibiotic at this time** | 18 | 75.0 |
| No antibiotic at this time; continue to investigate other possible causes of the arthritis | 2 | 8.3 |
| No antibiotic at this time; refer patient to a specialist | 2 | 8.3 |
| No antibiotic at this time; further testing for Lyme disease now | 2 | 8.3 |

***Correct response*

Q13. What is the CDC recommended treatment for adults diagnosed with **anaplasmosis**?  Select all that apply:

| **Response Option** | **N** | **%** |
| --- | --- | --- |
| Doxycycline for 10 -14 days** | 20 | 83.3 |
| Atovaquone + azithromycin for 7 -10 days | 4 | 16.7 |
| Clindamycin + quinine for 7-10 days | 2 | 8.3 |
| Amoxicillin for 10-14 days | 2 | 8.3 |

***Correct response*

Q14. What is the CDC recommended treatment for adults diagnosed with **babesiosis**? Select all that apply:

| **Response Option** | **N** | **%** |
| --- | --- | --- |
| Doxycycline for 10-14 days | 12 | 50.0 |
| Atovaquone + azithromycin for 7-10 days** | 10 | 41.7 |
| Clindamycin + quinine for 7-10 days** | 9 | 37.5 |
| Amoxicillin for 10-14 days | 2 | 8.3 |

***Correct response*

| **CDC-Recommended Treatments** | CDC-Recommended Treatments | |
| --- | --- | --- |
|  | Atovaquone + azithromycin for 7-10 days | Clindamycin + quinine for 7-10 days |
| Atovaquone + azithromycin for 7-10 days | 3 (12.5%) | 7 (29.2%) |
| Clindamycin + quinine for 7-10 days | 7 (29.2%) | 2 (8.3%) |
| *Total correct response: 12 (50.00%)* | | |

***Perceptions and Experiences***

Q15. To what extent do you agree with the following statements:

| **Statement** | **Response Option (N, %)** | | |
| --- | --- | --- | --- |
|  | **Disagree** | **Neutral** | **Agree** |
| I find laboratory results for tick-borne diseases hard to interpret | 7 (29.2) | 10 (41.7) | 7 (29.2) |
| I find laboratory results hard to interpret for Lyme disease specifically | 11 (45.8) | 7 (29.2) | 6 (25.0) |
| Patients question my knowledge regarding tick-borne diseases | 7 (29.2) | 6 (25.0) | 11 (45.8) |
| Patients do not understand me when I explain tick-borne disease diagnosis | 11 (45.8) | 4 (16.7) | 9 (35.5) |
| Patients do not trust my guidance regarding the treatment of tick-borne diseases | 11 (45.8) | 6 (25.0) | 7 (29.2) |

Q16. To what extent do you agree with the following statements:

| **Statement** | **Response Option (N, %)** | | |
| --- | --- | --- | --- |
|  | **Disagree** | **Neutral** | **Agree** |
| I am confident in my ability to discuss information about tick-borne diseases with patients | 2 (8.3) | 10 (41.7) | 12 (50.0) |
| I am confident in my ability to address misinformation about tick-borne diseases | 3 (12.5) | 12 (50.0) | 9 (37.5) |
| I am confident in my ability to address misinformation about Lyme disease specifically | 2 (8.3) | 12 (50.0) | 10 (41.7) |

Q17. How frequently do you encounter the following situations when discussing the use of antibiotics to treat tick-borne disease?

| **Statement** | **Response Option (N, %)** | | |
| --- | --- | --- | --- |
|  | Rarely | About half the time | Most of the time |
| Patients refuse to take the antibiotics I prescribe | 22 (91.7) | 2 (8.3) | 0 (0.0) |
| Patients request a shorter course of antibiotics than I am comfortable giving them | 22 (91.7) | 1 (4.2) | 1 (4.2) |
| Patients accept the antibiotic treatment plan I give to them* | 2 (8.37 | 4 (17.4) | 17 (73.9) |
| Patients request a longer course of antibiotics than I am comfortable giving them | 11 (45.8) | 9 (37.5) | 4 (16.7) |
| Patients try to negotiate with me on the length of their antibiotic course | 13 (54.2) | 7 (29.2) | 4 (16.7) |
| Patients request an alternative medicine form of treatment | 16 (66.7) | 4 (16.7) | 4 (16.7) |
| *1 non-response, total responses: N= 23 | | | |

***Tools and Resources***

Q18. To what extent do you agree with the following statements:

| **Statement** | **Response Option (N, %)** | | |
| --- | --- | --- | --- |
|  | **Disagree** | **Neutral** | **Agree** |
| I use educational tools with my patients to help them better understand tick-borne diseases | 4 (16.7) | 8 (33.3) | 12 (50.0) |
| Current resources available to me for patient education are sufficient | 6 (25.0) | 8 (33.3) | 10 (41.7) |

Q19. How often do you use the following resources to **educate patients** about tick-borne diseases?

| **Statement** | **Response Option (N, %)** | | | |
| --- | --- | --- | --- | --- |
|  | **Academic Medical Center** | | | **Small town Community** |
|  | **Never** | **Sometimes** | **Often** | **Yes/No*** |
| Resources from the local health department | 11 (61.1) | 6 (33.3) | 1 (5.6) | 1 (16.7) |
| Resources from the state health department | 12 (66.7) | 6 (33.3) | 0 (0.0) | 1 (16.7) |
| US Centers for Disease Control and Prevention website | 5 (27.8) | 10 (55.6) | 3 (16.7) | 4 (66.7) |
| US Centers for Disease Control and Prevention Tick-Borne Disease Handbook | 7 (38.9) | 9 (50.0) | 2 (11.1) | 1 (16.7) |
| Medscape** 1 no response (N = 17) | 13 (76.5) | 4 (23.5) | 0 (0.0) | 1 (16.7) |
| Up to Date** 2 no responses (N = 16) | 3 (18.8) | 5 (31.3) | 8 (50.0) | 6 (100.0) |
| Medical or public health journals** 2 no responses (N = 16) | 9 (56.3) | 5 (31.3) | 2 (12.5) | 1 (16.7) |
| Other (specify)^‡^ (N= 6) | 5 (83.3) | 0 (0.0) | 1 (16.7) | 2 (33.3) |
| * Small-town community respondents were asked to indicate use (Yes/No) rather than rate frequency of use. Their responses are separated from those of the academic medical center participants (Small-town N=6, Academic medical center N=18 unless otherwise specified). | | | | |
| ^‡^ Academic medical center written responses include: “My ID Docs”  ^‡^ Small-town community written responses include: “Local specialists” | | | | |

Q20. What do you like about the patient education materials you use? Select all that apply:

| **Response Option** | **N** | **%** |
| --- | --- | --- |
| Easy to Access*/Readily Available** | 16 | 66.7 |
| Easy to understand | 15 | 62.5 |
| Accurate information | 14 | 58.3 |
| Regularly updated*/Most up-to-date information** | 10 | 41.7 |
| Visually appealing | 5 | 20.8 |
| Easy to share with patients | 11 | 45.8 |
| Easy to print | 9 | 37.5 |
| Other (specify) | 0 | 0.0 |
| N/A - I do not use tick-borne illness patient educational materials* | 3 | 12.5 |
| * Wording used for academic medical center  ** Wording used for small-town community | | |

Q21. Would you like additional educational resources on tick-borne diseases for your patients?

| **Response Option** | **N** | **%** |
| --- | --- | --- |
| Yes | 20 | 83.3 |
| No | 4 | 16.7 |

Q22. What format would you prefer for educational resources for patients? Select all that apply:

| **Response Option** | **N** | **%** |
| --- | --- | --- |
| Posters | 2 | 8.3 |
| Flyers/pamphlets | 16 | 66.7 |
| Online resources | 13 | 54.2 |
| Other (specify) | 0 | 0.0 |

Q23. What topics should educational resources for patients to cover?

| **Response Option** | **N** | **%** |
| --- | --- | --- |
| Types of ticks in our area | 14 | 58.3 |
| How ticks transmit disease | 14 | 58.3 |
| Symptoms of tick-borne disease | 17 | 70.8 |
| Interpreting tests to diagnose tick-borne disease | 12 | 50.0 |
| Tick-borne disease treatment | 15 | 62.5 |
| Efficacy of tick-borne disease treatment | 14 | 58.3 |
| Other | 0 | 0.0 |

Q24. What do you think would make educational resources for patients more effective? [free-text responses]

- Simple language
- Simple algorithm
- Quick summary of information
- Pictures and diagrams

*Clinical Education Resources and Training Preferences*

Q25. To what extent do you agree with the following statements:

| **Statement** | **Response Option (N, %)** | | |
| --- | --- | --- | --- |
|  | **Disagree** | **Neutral** | **Agree** |
| I regularly look-up or research literature on tick-borne diseases. | 3 (12.5) | 10 (41.7) | 11 (45.8) |
| I have access to the resources I need to update my personal knowledge about tick-borne diseases. | 1 (4.2) | 4 (16.7) | 19 (79.2) |

Q26. How often do you use the following resources to access information on the management of tick-borne diseases?

| **Statement** | **Response Option (N, %)** | | | |
| --- | --- | --- | --- | --- |
|  | **Academic Medical Center** | | | **Small town Community** |
|  | Never | Sometimes | Often | Yes/No* |
| CME-accredited seminars | 10 (55.6) | 8 (44.4) | 0 (0.0) | 0 (0.0) |
| CME-accredited webinars | 16 (88.9) | 2 (11.1) | 0 (0.0) | 0 (0.0) |
| Guidelines from the Infectious Diseases Society of America | 6 (33.3) | 8 (44.4) | 4 (22.2) | 1 (16.7) |
| Guidelines from the International Lyme and Associated Diseases Society | 11 (61.1) | 7 (38.9) | 0 (0.0) | 0 (0.0) |
| Publications in medical journals | 4 (22.2) | 11 (61.1) | 3 (16.7) | 2 (33.3) |
| Centers for Disease Control and Prevention website (N=17) | 2 (11.8) | 9 (52.9) | 6 (35.3) | 4 (66.7) |
| Centers for Disease Control and Prevention Tick-Borne Disease Handbook | 9 (50.0) | 7 (38.9) | 2 (11.1) | 1 (16.7) |
| National Institutes of Health website | 9 (50.0) | 8 (44.4) | 1 (5.6) | 1 (16.7) |
| Medscape | 12 (66.7) | 6 (33.3) | 0 (0.0) | 1 (16.7) |
| Up to Date | 0 (0.0) | 5 (27.8) | 13 (72.2) | 6 (100.0) |
| Other (please specify)**: (N=5) | 5 (100.0) | 0 (0.0) | 0 (0.0) | 0 (0.0) |
| * Small-town community respondents were asked to indicate use rather than rate frequency of use. Their responses are separated from those of the academic medical center participants (Small-town N=6, Academic medical center N=18 unless otherwise specified). | | | | |
| ** Free-text responses include: “My ID Doctors”, “My hospital resources”, “none”, “N/A”, and “N/A” | | | | |

Q27. What is the number one way in which these resources can be improved? [free-text response]

- More visuals for interpreting data
- Ease of access; easy link on website for treatment and diagnosis; easy to access
- Clear, unambiguous recommendations

Q28. How likely are you to participate in a tick-borne disease training course that does **not** carry continuing education credit?

| **Response Option** | **N** | **%** |
| --- | --- | --- |
| Not likely | 8 | 33.3 |
| Somewhat likely | 12 | 50.0 |
| Very likely | 4 | 16.7 |

Q29. Please select the topics that would be **most useful to you** to include in a tick-borne disease training program: (select all that apply)

| **Response Option** | **N** | **%** |
| --- | --- | --- |
| Patient communication techniques on tick-borne diseases | 6 | 25.0 |
| Tick life cycle and biology | 2 | 8.3 |
| Species of ticks in your community | 2 | 8.3 |
| Epidemiological data for tick borne diseases in your community | 7 | 29.2 |
| Decision making around clinical management of tick-borne diseases | 18 | 75.0 |
| Use of decision support tools for tick borne disease treatment | 17 | 70.8 |
| Treatment for at-risk populations (e.g. outdoor workers) | 9 | 37.5 |
| Treatment for specific patients (pediatrics and contraindications) | 10 | 41.7 |
| Other (specify)** | 1 | 4.2 |
| **Other free-text response: “Diagnosis” | | |

Q30. Please rank the following training formats by your ability to access them, with the most accessible format ranked 1 and least accessible format ranked 5.

| **Response Option** | **Ranking Order (N, %)** | | | | |
| --- | --- | --- | --- | --- | --- |
|  | 1st | 2nd | 3rd | 4th | 5th |
| In-person seminar | 1 (4.2) | 2 (8.3) | 7 (29.2) | 10 (41.7) | 4 (16.7) |
| Printed materials | 6 (25.0) | 16 (66.7) | 1 (4.2) | 1 (4.2) | 0 (0.0) |
| Webinar | 2 (8.3) | 1 (4.2) | 13 (54.2) | 8 (33.3) | 0 (0.0) |
| Online materials | 15 (62.5) | 4 (16.7) | 3 (12.5) | 2 (8.3) | 0 (0.0) |
| Other | 0 (0.0) | 1 (4.2) | 0 (0.0) | 3 (12.5) | 20 (83.3) |

Q31. Please use the space below to share additional feedback regarding tick-borne disease training, communication, or resource needs that were not covered in this survey. [free-text response]

- Using test result visuals to help interpret
- None at this time; unsure; N/A
